# Convergence of virulence and antimicrobial resistance in increasingly prevalent *Escherichia coli* ST131 *papGII*+ sublineages

**DOI:** 10.1038/s42003-022-03660-x

**Published:** 2022-07-28

**Authors:** Michael Biggel, Pieter Moons, Minh Ngoc Nguyen, Herman Goossens, Sandra Van Puyvelde

**Affiliations:** 1 Laboratory of Medical Microbiology, Vaccine & Infectious Disease Institute, University of Antwerp, Antwerp, Belgium; 2 Cambridge Institute of Therapeutic Immunology & Infectious Disease (CITIID), Department of Medicine, University of Cambridge, Cambridge, UK; 3 Present Address: Institute for Food Safety and Hygiene, Vetsuisse Faculty, University of Zurich, Zurich, Switzerland

**Keywords:** Bacterial genes, Population genetics

## Abstract

*Escherichia coli* lineage ST131 is an important cause of urinary tract and bloodstream infections worldwide and is highly resistant to antimicrobials. Specific ST131 lineages carrying invasiveness-associated *papGII* pathogenicity islands (PAIs) were previously described, but it is unknown how invasiveness relates to the acquisition of antimicrobial resistance (AMR). In this study, we analysed 1638 ST131 genomes and found that *papGII*+ isolates carry significantly more AMR genes than *papGII*-negative isolates, suggesting a convergence of virulence and AMR. The prevalence of *papGII*+ isolates among human clinical ST131 isolates increased dramatically since 2005, accounting for half of the recent *E. coli* bloodstream isolates. Emerging *papGII*+ lineages within clade C2 were characterized by a chromosomally integrated *bla*CTX-M-15 and the loss and replacement of F2:A1:B- plasmids. Convergence of virulence and AMR is worrying, and further dissemination of *papGII*+ ST131 lineages may lead to a rise in severe and difficult-to-treat extraintestinal infections.

## Abstract (web summary)

Genomic analyses indicate that the ST131 lineage of E. coli, responsible for urinary and bloodstream infections globally, is evolving towards both increased virulence and increased resistance to antimicrobials.

## Introduction

A large proportion of human urinary tract and bloodstream infections are caused by a few globally dispersed *E. coli* clones, including sequence type (ST) 69, ST73, ST95, and ST131^1^. Despite its recent emergence, ST131 is the dominant multi-drug resistant clone among extraintestinal pathogenic *E. coli* (ExPEC) isolates today^2^. In particular, high rates of resistance to 3^rd^-generation cephalosporins and fluoroquinolones among ST131 isolates present a major public health risk, leading to its classification as a critical priority pathogen by the WHO^3^.

The ST131 population is phylogenetically divided into clades A, B, and C. Clade C can be further divided into subclades C0, C1, and C2. The latter two harbour chromosomal mutations in quinolone-resistance determining regions (QRDR) of *gyrA* and *parC*, conferring high-level fluoroquinolone resistance. The emergence of the most recent common ancestor of subclades C1 and C2 was dated to 1992, which coincided with increased fluoroquinolone use worldwide^4–6^. The expansion of subclade C2, which represents the bulk of the current ST131 pandemic, is assumed to have been driven by the acquisition of a specific IncFII plasmid (plasmid multilocus sequence type [pMLST] F2:A1:B-) carrying *bla*CTX-M-15 (extended-spectrum beta-lactamase [ESBL]). Isolates of subclade C1 frequently harbour the ESBL-encoding genes *bla*CTX-M-14 or *bla*CTX-M-27, whereas ESBLs are less prevalent in clade A and B^4–9^.

ST131 colonizes the human gastrointestinal tract as a commensal but causes mild to severe infections in the urinary tract including pyelonephritis and urosepsis. Specific ST131 sublineages are also overrepresented among asymptomatic bacteriuria^10,11^, which may be explained by varying underlying virulence profiles in these sublineages. Irrespective of their clade affiliation, most ST131 isolates carry mobile genetic elements encoding synthesis of the siderophores aerobactin (*iuc*) and yersiniabactin (*ybt*), two important factors promoting extraintestinal colonization^12,13^. Other virulence factors such as *hly* (hemolysin), *iro* (salmochelin siderophore), *agg* (aggregative adherence fimbriae AAF), or *papGII* (P fimbrial tip adhesin variant PapGII) are restricted to specific ST131 sublineages^11,14,15^. Of particular significance is *papGII*, which was recently identified as a key determinant of invasive uropathogenic *E. coli* (UPEC) in infection experiments and genome-wide association studies^11,16,17^. Approximately 60% of *E. coli* isolates from invasive urinary tract infections (i.e., pyelonephritis or bacteremia with a urinary portal of entry) carry *papGII*, while the gene is less common among isolates from patients with cystitis or asymptomatic bacteriuria^11^. PapGII drives inflammation and renal tissue damage through transcriptional activation of signalling pathway genes in kidney cells, resulting in kidney and bloodstream infections^16^. Overall, approximately half of all *E. coli* bacteremia cases are associated with an entry through the urinary tract^18–20^.

ST131 comprises multiple sublineages that independently acquired *papGII*-containing pathogenicity islands (*papGII* PAIs)^11,21^. Currently, it is unknown how the antimicrobial resistance gene (ARG) content relates to these *papGII*-containing (*papGII*+) lineages. Dissemination of highly resistant and more invasive lineages would aggravate the global burden of already difficult-to-treat infections caused by *E. coli*.

In *E. coli*, virulence and AMR are frequently encoded by mobile genetic elements such as plasmids, genomic islands (PAIs and resistance islands [REIs]), bacteriophages, or transposons^22–24^. Relative high rates of acquisitions of mobile genetic elements with a highly dynamic accessory genome^9^ make *E. coli* ST131 an interesting model to examine the co-evolution of AMR and virulence. In this study, we investigate the population structure, resistome, and distribution of *papGII* in ST131 using 1638 publicly available genomes of human isolates. Our results reveal significant evolutionary changes and a genetic convergence of virulence and AMR in increasingly prevalent *papGII*+ sublineages of ST131.

## Results

### Isolate collections and resistome

Publicly available genomes of 1638 *E. coli* ST131 isolates were analysed in this study. These included 1538 whole-genome draft assemblies from 11 collections and 100 high-quality reference assemblies (Supplementary Data 1). The isolates originated from human bloodstream infections (*n* = 843), urinary tract infections (*n* = 306), feces (*n* = 83), and other (*n* = 9) or unknown (*n* = 397) clinical sources, and were isolated between 2001 and 2017 in Europe, North America, Asia, and Oceania (Table 1). In eight of the 11 source studies (comprising 1148 isolates), isolates were specifically selected for being ESBL-producing. Genome sizes ranged from 4.69 Mb to 5.73 Mb, and all assemblies passed quality control (N50 > 45 kb, >99% completeness).

**Table 1:** Isolate collections included in this study.

Collection	No. ST131 isolates	Clinical source	Pre-selection of isolates	Time and country of isolation	NCBI Bioproject accession	Source study
Birgy	94	fUTI	ESBL+	2014–2016, France	PRJNA551371	Birgy et al.^66^
Froeding	122	BSI	ESBL+	2012–2015, Sweden	PRJNA612606	Froeding et al.0^8^
Harris	43	BSI	ESBL+	2014–2015, Australia, New Zealand, Singapore	PRJNA398288	Harris et al.^67^
Kallonen	221	BSI	-	2001–2012, UK	PRJEB4681	Kallonen et al.^14^
Kossow	71	feces	ESBL+	2015–2016, Germany	PRJEB23208	Kossow et al.^68^
Ludden	90	urine (*n* = 80), BSI (*n* = 4), sputum (*n* = 4), feces (*n* = 2)	ESBL+ (partly)	2005–2011, Ireland	PRJEB2974	Ludden et al.^6^
MacFadden	87	BSI	–	2010–2015, Canada	PRJNA521038	MacFadden et al.^69^
Miles-Jay	130	urine (*n* = 123), BSI (*n* = 4), bone (*n* = 3)	ESBL+, *fimH*30	2009–2013, US	PRJNA578285	Miles-Jay et al.^70^
Roer	259	BSI	ESBL+	2014–2015, Denmark	PRJEB20792	Roer et al.^71^
Septicoli	82	BSI	–	2016–2017, France	PRJEB35745	De Lastours et al.^72^
SoM-study	339	n.r.	ESBL+	2011–2014, Netherlands	PRJEB15226	Kluytmans-van den Bergh et al.^73^

*fUTI* febrile urinary tract infection, *BSI* bloodstream infection, *n.r.* not reported, *ESBL+* extended-spectrum beta-lactamase-producing *E. coli*

**Fig. 1:**
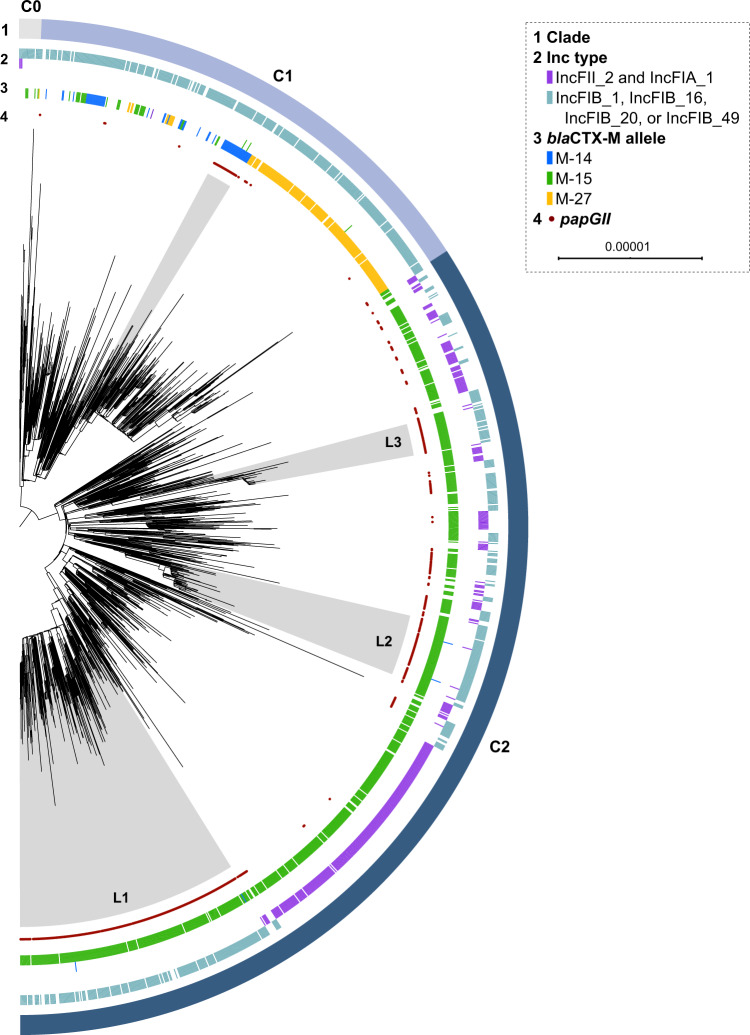
Phylogenetic tree of ST131 clade C. Maximum-likelihood phylogenetic tree of 1338 clade C0, C1, and C2 isolates based on 10,904 variable sites in a 2.5 Mb core genome alignment. Each isolate is annotated with ST131 subclade affiliation (ring 1), presence of selected IncF plasmid replicon types (pMLST; ring 2), *bla*CTX-M allele (ring 3), and *papGII* gene (ring 4). ST131 *papGII*-containing sublineages discussed in the text are shaded in grey. Those of clade C2 are annotated with L1, L2, and L3. The scale bar indicates the number of substitutions per site. The tree was visualized using iTOL^74^. Supplementary Fig. 2 shows this tree with additional information.

**Fig. 2:**
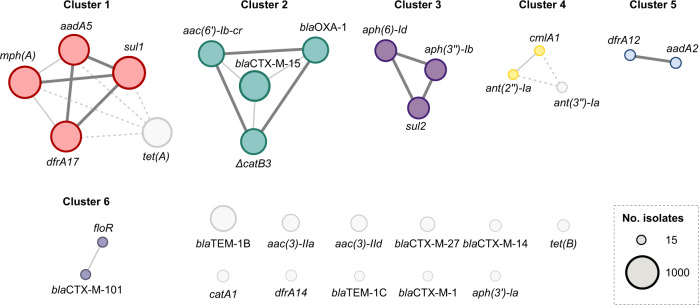
Co-occurrence network graph of antimicrobial resistance genes (ARGs). ARGs occurring in at least 15 isolates are shown. Circle sizes correspond to the number of occurrences. Co-occurring genes are connected according to the co-occurrence level in thick (Jaccard distance [JD] < 0.15 [commonly co-occurring]), thin (JD 0.15–0.3), or dashed (JD 0.3–0.5) lines.

Overall, 102 distinct ARGs were identified in the ST131 isolates (Supplementary Data 2). In agreement with previous studies^9^, clade C2 was strongly associated with the presence of *bla*CTX-M-15 (found in 89% of C2 isolates), while the presence of ESBL genes (including *bla*CTX-M-1, M-14, M-15, M-27, and M-101) in other clades was more variable and often confined to specific sublineages (Fig. 1, Supplementary Fig. 1, Supplementary Table 1). Multiple ARGs showed co-occurrence, suggesting co-acquisition, co-location, and co-selection during antibiotic exposure (Fig. 2). Eleven ARGs typically (in 73–100% of all individual occurrences) co-occurred in one of three clusters: (1) *aadA5*, *dfrA17*, *mph(A)*, and *sul1* (Fig. 2 cluster 1); (2) *aac(6*′*)-lb-cr*, *bla*CTX-M-15, *bla*OXA-1, and (Δ)*catB3* (Fig. 2 cluster 2); and (3) *aph(3″)-Ib*, *aph(6)-Id*, and *sul2* (Fig. 2 cluster 3). The eleven ARGs individually accounted for 71.3% of the entire ST131 ARG content. Cluster 1 was common in clade A (42.5% of all A isolates), C1 (54.7%), and C2 (56.8%), but uncommon in clade B (4.8%). Cluster 2 occurred almost exclusively in clade C2 (70.0% of all C2 isolates) and in ≤ 2% of clade A, B, or C1 isolates. Cluster 3 was common in clade A (47.1%), B (31.0%), and C1 (57.4%) and uncommon in C2 (4.4%). None of the clusters showed a clear co-occurrence pattern (i.e., Jaccard distance <0.3) with any of the 55 detected alleles from the IncF plasmid replicon family or 41 plasmid replicon types from other families.

### Sublineages with papGII+ isolates are associated with increased AMR

Phylogenetic analyses showed one *papGII*+ sublineage each in clades A, B, and C1, consistent with previous work^11^. Most *papGII*+ isolates (444/547, 81.1%) belonged to clade C2, which harboured multiple *papGII*+ sublineages, including three major *papGII*+ sublineages (named L1, L2, and L3; Fig. 1 and Supplementary Fig. 1). The largest *papGII*+ sublineage (clade C2 sublineage L1) comprised almost half of all *papGII*+ isolates (230/547, 42.0%). High-quality assemblies and contig homologies confirmed the predominance of type III *papGII* PAIs within the ST131 population, but type II and type IV *papGII* PAIs were also identified (Supplementary Table 2, Supplementary Fig. 3, Supplementary Fig. 4).

*papGII*+ isolates presented with an increased ARG content: on average, *papGII*+ isolates harboured 8.7 ARGs (median 9; SD 3.6) versus 6.3 ARGs (median 7; SD 3.7) among *papGII*-negative isolates. The positive association between *papGII* presence and ARGs was found to be significant (*P*_*adj*_ < 0.05, Mann-Whitney U test) within each of the ST131 clades A, B, C1, and C2 and across different isolation time intervals (Supplementary Table 3). This significant association was also found irrespective of whether isolates were pre-selected for being ESBL-producing and among urinary and blood isolates. The increased ARG content was not significant among faecal isolates, for which limited data was available. This association was confirmed using an extended dataset, comprising assemblies of the main dataset and 3,608 additional assemblies of human ST131 isolates from EnteroBase. The extended dataset showed that *papGII*+ isolates from bloodstream infections carry similar numbers of ARGs than *papGII*+ isolates of urinary or faecal origin. Regardless of their source, significantly more ARGs were found among *papGII*+ isolates than among *papGII*-negative isolates (Supplementary Table 4). As the acquisition of *papGII* occurs via PAIs^25^ and acquisition of ARGs predominantly via plasmids^24^, virulence and AMR acquisition likely occurred independently. Among the 30 resolved *papGII*+ PAIs from high-quality assemblies, only one contained ARGs within the same PAI (Supplementary Table 2).

The difference in the ARG content between *papGII*+ and *papGII*-negative isolates could not be attributed to one specific ARG or AMR class. When stratified by clade, different ARGs were significantly (*P*_*adj*_ < 0.05, Fisher’s exact test) associated with *papGII*+ isolates, including those conferring resistance against 3^rd^-generation aminoglycosides, cephalosporins, fluoroquinolones, sulfamethoxazole, and trimethoprim (Table 2, Supplementary Table 5, Supplementary Fig. 5).

**Table 2:** Acquired antimicrobial resistance genes (ARGs) significantly (*P*_*adj*_^a^ < 0.05) associated with *papGII*-containing (*papGII*+) versus *papGII*-negative isolates.

ARG	Resistance class	ARG co-occurrence cluster	Prevalence *papGII*+ isolates (*n* = 547)	Prevalence *papGII*-negative isolates (*n* = 1091)	Odds ratio (95% CI)
*aac(3)-IIa*	Aminoglycoside	–	238 (43.5%)	85 (7.8%)	9.1 (6.9–12.0)
*aac(3)-IId*	Aminoglycoside	–	136 (24.9%)	160 (14.7%)	1.9 (1.5–2.5)
*aac(6’)-Ib-cr*	Aminoglycoside, Fluoroquinolone	Cluster 2	375 (68.6%)	320 (29.3%)	5.3 (4.2–6.6)
*aadA2*	Aminoglycoside	Cluster 5	45 (8.2%)	28 (2.6%)	3.4 (2.1–5.5)
*bla*CTX-M-15	Beta-lactam (ESBL)	Cluster 2	435 (79.5%)	455 (41.7%)	5.4 (4.3–6.9)
*bla*CTX-M-27	Beta-lactam (ESBL)	–	28 (5.1%)	188 (17.2%)	0.26 (0.17–0.39)
*bla*CTX-M-101	Beta-lactam (ESBL)	Cluster 6	26 (4.8%)	1 (0.1%)	54.4 (7.4–401.9)
*bla*OXA-1	Beta-lactam	Cluster 2	374 (68.4%)	324 (29.7%)	5.1 (4.1–6.4)
*catA1*	Phenicol	–	78 (14.3%)	16 (1.5%)	11.2 (6.5–19.3)
(Δ)*catB3*	Phenicol	Cluster 2	372 (68.0%)	318 (29.1%)	5.2 (4.1–6.5)
*dfrA12*	Trimethoprim	Cluster 5	45 (8.2%)	21 (1.9%)	4.6 (2.7–7.7)
*dfrA14*	Trimethoprim	–	34 (6.2%)	27 (2.5%)	2.6 (1.6–4.4)
*floR*	Phenicol	Cluster 6	25 (4.6%)	6 (0.5%)	8.7 (3.5–21.2)
*tet(B)*	Tetracycline	–	56 (10.2%)	43 (3.9%)	2.8 (1.8–4.2)

^a^Fisher’s exact text, Bonferroni corrected for the overall number of identified ARGs (*n* = 102).

*papGII*+ sublineages in clades A, B, and C1 were marked by the presence of *bla*CTX-M-27, *bla*CTX-M-101, and *bla*CTX-M-14, respectively (Fig. 1, Supplementary Fig. 1). In clade C2, most isolates (88.8%) harboured *bla*CTX-M-15 irrespective of *papGII* presence. Because *papGII*+ isolates were enriched in clade C, a higher proportion of *papGII*+ isolates contained >3 chromosomal mutations in QRDRs (88.1% vs 77.5% of *papGII*-negative isolates, *P* < 0.001 [Fisher’s exact test], OR = 2.2 [95% CI 1.6–2.9]). Overall, 85.2% of *papGII*+ isolates were predicted to be resistant to both ciprofloxacin (mediated by QRDR mutations or *aac(6’)-Ib-cr*) and 3^rd^ generation cephalosporins (mediated by ESBL) compared to 59.8% of *papGII*-negative isolates (*P* < 0.001 [Fisher’s exact test], OR = 3.9 [95% CI 3.0–5.0]). In the three isolate collections originally not pre-selected for ESBL-producing *E. coli*, 72.4% of *papGII*+ isolates were predicted to be resistant against both ciprofloxacin and 3^rd^ generation cephalosporins, compared to 26.1% of *papGII*-negative isolates (*P* < 0.001 [Fisher’s exact test], OR 7.4 [95% CI 4.2–13.0]).

### C2 papGII+ sublineages are increasingly prevalent and frequently harbour chromosomal blaCTX-M-15

**Fig. 3:**
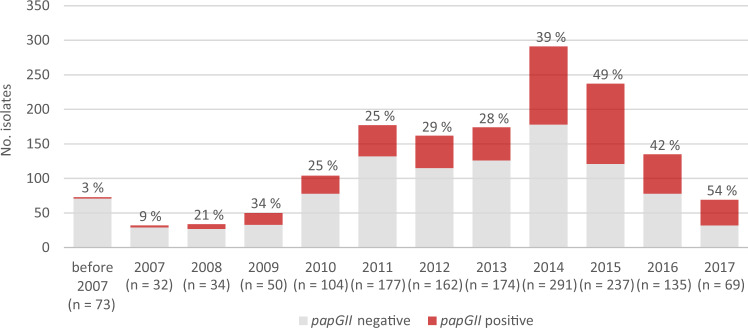
Proportion of *papGII*-containing isolates in the ST131 population over time. 1538 isolates from the 11 investigated collections were analysed. The proportion of *papGII-*containing isolates per year is coloured in red and the percentage is indicated above each bar. The total number of isolates per year is given in brackets. A plot showing the cumulative proportion of *papGII*-containing isolates is shown in Supplementary Fig. 6.

Before 2007, *papGII* was rarely identified in ST131 isolates. Since then, the proportion of *papGII*+ isolates in the investigated ST131 population has increased and accounted for approximately 50% of the most recently (2015–2017) collected isolates (Fig. 3). The gradual increase in the prevalence of *papGII* since approximately the year 2005 was confirmed in the validation dataset of 3,608 ST131 genomes from human isolates available on EnteroBase. In this dataset, the proportion of *papGII*+ isolates increased from 8% before 2007 to 28–35% in recent years (2016–2019) among all human isolates and to 46–61% among human blood isolates (Supplementary Fig. 7). The proportion was strongly influenced by the isolates’ clinical source with *papGII* being more frequently detected in blood isolates (475/789, 39.8%) than in urine/UTI-associated isolates (255/936, 27.2%; *P* < 0.001 [Fisher’s exact test]; OR 4.0 [95% CI: 3.3–4.9]) or faecal isolates (65/513, 12.7%; *P* < 0.001; OR 10.4 [95% CI:7.7–14.0]).

Clade C2 is a major cause of the ongoing ExPEC pandemic^6–8^ and the success of early clade C2 sublineages has been attributed in part to the stable maintenance of pMLST F2:A1:B- plasmids containing *bla*CTX-M-15^5,26^. Here we observed that the plasmid replicon profile in clade C2 differed between *papGII*+ and *papGII*-negative isolates. Among clade C2 isolates, putative F2:A1:B- plasmids (indicated by the presence of both pMLST alleles FII_2 and FIA_1 in an assembly) were found in 327 (69.7%) *papGII*-negative isolates but in only 26 (5.9%; OR 37.0 [95% CI: 23.8–57.6]) *papGII*+ isolates (Fig. 1). In contrast, clade C2 *papGII*+ isolates were associated with various IncFIB replicon types: 361 (81.3%) *papGII*+ isolates carried FIB_1, FIB_16, FIB_20, or FIB_49, versus 85 (18.1%; OR 19.6 [95% CI: 14.1 – 27.5]) *papGII*-negative isolates. The three major clade C2 *papGII*+ sublineages L1, L2, and L3 were associated with FIB_1, FIB_49, and FIB_1, respectively (Table 3, Supplementary Fig. 2). More specifically, L1 isolates predominantly contained pMLST alleles FII_31 (50% of all L1 isolates), FII_36 (41%), FIA_4/FIA_20 (79%), and FIB_1 (83%); L2 isolates contained FII_48 (86%), FIA_1 (100%), FIA_6 (100%), and FIB_49 (97%); and L3 isolates contained FII_36 (85%) and FIB_1 (82%). *bla*CTX-M-15 was identified in the majority of C2 isolates irrespective of the present plasmid replicon type (in 302/353 [85.6%] isolates carrying FII_2 and FIA_1; and in 414/446 [92.8%] isolates carrying FIB_1, FIB_16, FIB_20, or FIB_49). These observations imply that in clade C2 *papGII*+ isolates, ESBLs are generally not located on F2:A1:B- plasmids.

**Table 3:** Characteristics of dominant ST131 *papGII*-containing (*papGII*+) sublineages.

*papGII*+ sublineage	No. isolates	*papGII*+ PAI type	Dominant IncF pMLST alleles	Dominant ESBL-encoding genes	Dominant genetic context of ESBL-encoding genes (locus; evidence)	Representative isolate
*papGII*+ sublineage clade A	24	III	FII_29, FIB_10	*bla*CTX-M-27 (*n* = 24)	chromosomal (near *gspD*; found in 22/24 isolates with resolved context)	A17EC0155 (GCF_021133255.1)
*papGII*+ sublineage clade B (*fimH*27)	39	II and III	FII_1, FIB_63	*bla*CTX-M-101 (*n* = 27), *bla*CTX-M-15 (*n* = 5)	*not resolved*	RDE6 (GCA_013027405.1)
*papGII*+ sublineage clade C1	27	III	FII_1, FIA_2, FIA_6, FIB_20	*bla*CTX-M-14 (*n* = 27)	chromosomal (near *cmtA*; found in 12/14 isolates with resolved context)	222A118 (GCF_020230335.1)
*papGII*+ sublineage clade C2 L1	236	III	FII_31, FII_36, FIA_20/FIA_4, FIB_1	*bla*CTX-M-15 (*n* = 225)	subbranch L1b (*n* = 52): *not resolved*, subbranch L1a (*n* = 184): chromosomal (near *metG*; found in 10/10 isolates with resolved context; 184 isolates with disrupted DUF4132 region)	US02 (GCA_014140815.1)
*papGII*+ sublineage clade C2 L2	65	IV	FII_48, FIA_1, FIA_6, FIB_49	*bla*CTX-M-15 (*n* = 65)	chromosomal (into *mppA*; found in 59/61 isolates with resolved context; 64 isolates with disrupted *mppA*)	2/0 (GCA_003856635.1)
*papGII*+ sublineage clade C2 L3	34	III	FII_36, FIB_1	*bla*CTX-M-15 (*n* = 34)	chromosomal (into *ydhS*; found in 14/18 isolates with resolved context)	ESBL41 (GCF_007109465.1)

**Fig. 4:**
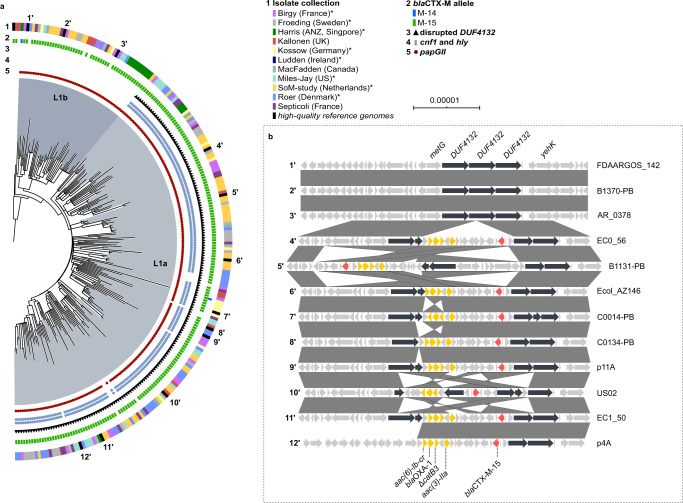
Phylogeny and characteristics of the dominant ST131 *papGII*+ sublineage L1 (clade C2). **a** Maximum-likelihood phylogenetic tree of 236 isolates of the clade C2 branch encompassing the dominant *papGII*+ sublineage L1 (including 6 *papGII*-negative isolates) and EC958 (clade C2) as outgroup. The tree is based on 2,790 variable sites in a 3.4 Mb core genome alignment. The two major subbranches (L1a and L1b) of L1 are shaded in different colours. Each isolate is annotated with the source collection (ring 1), *bla*CTX-M allele (ring 2), disruption of the *DUF4132* region (ring 3), and presence of *hly*, *cnf1* (ring 4), and *papGII* (ring 5). Collections that consisted only of ESBL isolates are labelled with an asterisk in the legend. The number of substitutions per core genome alignment site is indicated by the scale bar. The tree was visualized using iTOL^74^. Numbers at the outermost ring refer to the 12 isolates with high-quality assemblies and resolved *DUF4132* region, which are illustrated in panel b. Sublineage L1 was unambiguously defined within the ST131 population by a distinct *mtlD* allele (encoding a mannitol dehydrogenase family protein; 96% sequence identity to the *mtlD* allele of other ST131 isolates; Genbank accession number TLH04005.1) **b** Genetic context of the *DUF4132* region in high-quality assemblies from 12 isolates of *papGII*+ sublineage L1. All 9 isolates of subbranch L1a (isolates 4′ to 12′) harboured Tn*MB1860* with the resistance genes *aac(3)-IIa*, *aac(6’)-Ib-cr*, *bla*OXA-1, *bla*CTX-M-15, and Δ*catB3* inserted into the chromosomal *DUF4132* region. Vertical boxes between sequences indicate shared homologies (100% identity). Sequence comparisons were performed using EasyFig^64^.

Isolates from the three major *papGII*+ sublineages in clade C2 typically harboured *bla*CTX-M-15 integrated into the chromosome (Table 3). In the largest ST131 *papGII*+ sublineage L1 (clade C2; 236 isolates, including 6 *papGII*-negative isolates), a subbranch (L1a, 184 isolates) was characterized by a *bla*CTX-M-15-containing transposon Tn*MB1860* integrated into the chromosomal *metG/DUF4132/yeh* region (Fig. 4). Tn*MB1860* was previously described for a clade C2 isolate by Shropshire et al.^27^ and additionally contains *aac(3)-IIa*, *aac(6’)-Ib-cr*, *bla*OXA-1, a truncated (Δ)*catB3*, and *tmrB*. Unlike isolates of subbranch L1b, L1a isolates also contained virulence-associated genes encoding the toxins hemolysin (*hly*) and cytotoxic necrotizing factor 1 (*cnf1*) on their *papGII*+ PAI. Four isolates that fell into the L1a lineage lacked *papGII*, *hly*, *cfn1*, and *ucl*, suggesting that they lost the entire PAI. L1 consisted of isolates from all collections except one (Ludden), suggesting global dissemination (Supplementary Fig. 2).

The second-largest ST131 *papGII*+ sublineage L2 (clade C2; 64 isolates, including 6 *papGII*-negative isolates) harboured a *bla*CTX-M-15-containing transposon chromosomally integrated into *mppA* (encoding a murein peptide-binding protein), as described previously by Ludden et al.^6^. Sublineage L2 consisted mostly of isolates from a single outbreak-associated collection obtained in Ireland (Supplementary Fig. 2).

The third-largest ST131 *papGII*+ sublineage L3 (clade C2; 34 isolates) was epidemiologically more diverse with isolates originating from Asia, Europe, and North America (Supplementary Fig. 2). In 14 isolates, *bla*CTX-M-15 was chromosomally integrated into *ydhS* (encoding a putative oxidoreductase); in 4 isolates *bla*CTX-M-15 was integrated at other chromosomal positions (near *aphA*, *ghrB*, *ubiX*, or into prophage mEp460); in one isolate it was located on a plasmid; and in the remaining 15 isolates, the *bla*CTX-M-15 genetic context could not be resolved from short-read assemblies. Also in *papGII*+ sublineages of ST131 clades A and C1, *bla*CTX-M genes were often chromosomally integrated (Table 3). Incomplete assemblies from short-read data did however not allow a systematic evaluation of *papGII*+ versus *papGII*-negative isolates.

## Discussion

Our genomic study of global public ST131 data suggests a convergence of virulence and AMR in increasingly prevalent *papGII*+ *E. coli* ST131 sublineages. ARGs enriched in *papGII*+ isolates included those conferring resistance against fluoroquinolones, 3^rd^-generation cephalosporins, aminoglycosides, and trimethoprim/sulfamethoxazole, which are important treatment options for urinary tract infections and bacteraemia. Most *papGII*+ sublineages expanded after the year 2005 within the multi-drug resistant clade C2, implying that PAIs harbouring *papGII*+ were acquired after AMR determinants such as the clade C-specific QRDR mutations and *bla*CTX-M-15. However, also within this clade C2, we observed higher levels of AMR in *papGII*+ isolates compared to *papGII*-negative isolates, suggesting a further synergy between AMR and virulence. We assume that virulence genes may contribute to the maintenance and further acquisition of ARGs by causing more severe disease which needs more extensive treatment. UPEC typically reside in the gut implying that antibiotic treatment for UTI may create evolutionary pressure both in the gut and at the site of infection^23,28^. Increased AMR may hence result in prolonged extraintestinal colonization, intestinal blooms, and eventually enhanced dissemination of specific UPEC clones^29^. The observed convergence of AMR and virulence in ST131 may not be generalized to other *E. coli* lineages. For example, the pandemic *E. coli* lineages ST73 and ST95 frequently cause extraintestinal infections but AMR levels remain relatively low without leading to a displacement by more resistant lineages^11,14,30^. This might be explained by differences between lineages in their ability to acquire genes and integrate them into regulatory and functional processes, or by their different lifestyles and occupation of niches^31,32^.

While the success of early clade C2 sublineages was partly attributed to the maintenance of pMLST F2:A1:B- plasmids containing *bla*CTX-M-15^5^, we observed that the more recently emerged *papGII*+ sublineages within clade C2 were characterized by the presence of various IncFIB plasmids and chromosomally encoded *bla*CTX-M-15. Transpositions of *bla*CTX-M-15 from the plasmid to the chromosome of clade C2 isolates have been described in multiple prior studies^5,6,9,27,33,34^. The chronological order of AMR and *papGII* acquisitions could not be elucidated with confidence from our data. Conceivably, (i) transposition of *bla*CTX-M-15 from an F2:A1:B- plasmid to the chromosome was followed by (ii) the loss of F2:A1:B- plasmids, (iii) the acquisition of FIB plasmids, and (iv) the acquisition of *papGII*+ PAIs. Varying selective advantages of plasmids or plasmid-PAI incompatibilities may underly the co-presence of *papGII*+ and IncFIB in clade C2. Specific plasmids may for example be involved in the horizontal co-transfer of *papGII*+ PAIs: *E. coli* PAIs typically lack mobilization and transfer genes, but conjugative (co-)transfer of UPEC PAIs was previously shown in vitro using helper plasmids^35,36^. A complex plasmid-island interaction is for example known for *S. enterica*, where the mobilizable resistance island SGI1 is incompatible with IncC/A plasmids, but relies on those for propagation^37^.

The increasing dominance of *papGII*+ ST131 strains has been reported before. Royer et al.^21^ described an increase of *papGII*+ from 10 to 46% between 2005 and 2016 in ST131 bloodstream isolates from France. Kallonen et al.^14^ found an increase of *papG* from 8 to 44% between 2003 and 2012 in ST131 bloodstream isolates from England. Ludden et al. reported the displacement of a C1 sublineage by a C2 sublineage among residents of a long-term care facility in Ireland between 2005 and 2011^6^. This C2 sublineage carried a chromosomal *bla*CTX-M-15 and was here found to also harbour *papGII*.

Sublineage L1a was most abundant among the *papGII*+ ST131 sublineages. L1a was globally disseminated and characterized by (i) a PAI with *papGII*, *cnf1*, and *hly*, (ii) a chromosomal resistance cassette with *aac(3)-IIa*, *aac(6’)-Ib-cr*, *bla*OXA-1, and *bla*CTX-M-15, and (iii) frequent carriage of FIB_1 and FIA_4/20 plasmids. Isolates with these features were previously reported by Chen et al.^7^ among bacteremia isolates collected in 2015 in South East Asia and associated with increased virulence and AMR. Likewise, Pajand et al. described such clade C2 isolates among ST131 isolates from Iran^38^. An apparent stable integration of *bla*CTX-M-15 and *aac(6’)-Ib-cr* might have contributed to its success: in clade C2, the ciprofloxacin-inactivating AAC(6’)-Ib-cr was shown to confer a selective advantage in the presence of ciprofloxacin over isolates that contained QRDR mutations alone^39^.

A limitation of our study was that 8 of the 11 ST131 collections (1148 isolates) were pre-selected for ESBL-producing isolates, introducing sampling bias. Among those, ESBL genes were detected in 1062 (92.5%) isolates, compared to 146/390 (37.4%) isolates from the 3 remaining collections, suggesting that AMR (in particular ESBL prevalence) was overestimated here. We stratified the statistical analysis by pre-selection criteria to take this into account. In addition, with more than half of all isolates originating from bloodstream infections, *papGII*-containing isolates are likely also overrepresented in our data relative to the overall ST131 population. Furthermore, the investigated isolates were not available for phenotypic AMR validation. Sensitivities and specificities of AMR genotype-phenotype predictions in *E. coli* were previously estimated to be >95% and >90%, respectively, for most antibiotics^22^. Lastly, we were unable to determine the genomic location of most ARGs from the available short-read data. Long-read sequencing of more isolates would allow a better understanding of the observed association between *papGII*, chromosomal *bla*CTX-M elements, and specific plasmid replicons, but has not been employed for large collections like the ones studied here due to the high costs.

In conclusion, we describe the convergence of virulence and AMR in *papGII*+ ST131 sublineages, which is an important concept among emerging pathogens. A similar convergence of virulence and AMR in specific clones has been observed in other pathogens, including *K. pneumoniae*^40^, *S. enterica*^41^, and *S. aureus*^42^. UTIs caused by ST131 *papGII*+ strains presumably lead to more severe infections and are more challenging to treat with commonly used antibiotics, underlining the need for novel preventive and curative strategies to manage infections.

## Methods

### Bacterial genomes: main dataset

Genomes of 1638 *E. coli* ST131 isolates of human origin were included in this study. 1538 genomes originated from 11 publicly available collections. Inclusion criteria for collections were (i) a large number (>40) of ST131 genomes within a single collection, (ii) the availability of metadata, and (iii) a human source of isolation. In addition, 100 public high-quality assemblies (N50 > 1.5 Mb; derived from long-read sequencing) were included for the analyses of mobile genetic elements that could not be resolved in assemblies from short-read data. Assemblies were obtained from EnteroBase^43^ or NCBI and sequence types were confirmed with the Achtman scheme^43^ using mlst 2.19.0^44^. Quast v5.2.0^45^ and CheckM v1.1.3^46^ were used for the quality control of assemblies. Assemblies with N50 values of >40 kb were considered acceptable. Details on individual isolates including metadata, assembly methods, assembly metrics, and accession numbers are provided in Supplementary Data 1. For three of the 11 isolate collections, the isolates were annotated with the isolation time period instead of the precise isolation year (Roer: 2014–2015; Septicoli: 2016–2017; SoM study: 2011–2014). To determine trends in the ST131 population over time, those isolates were randomly assigned to years within the given period.

### Bacterial genomes: validation dataset

The prevalence of *papGII*-containing ST131 isolates over time and the association of *papGII* with different isolation sources were determined using a larger dataset of 3,608 *E. coli* ST131 genomes. This dataset comprises all ST131 assemblies available on EnteroBase (accessed on 07/01/2021)^43^ of isolates recovered from human samples (based on BioSample metadata) and annotated with a year of isolation. Assemblies already included in the main dataset and of low assembly quality (N50 < 40 kb) were excluded. Details on the included assemblies are provided in Supplementary Data 3.

### Phylogenetic analyses

Core-genome alignments were created using parsnp v1.2^47^ (default options) with chromosomal sequences of EC958 (for clade C, C2, or sublineage L1 alignments), E41-1 (for clade C1 alignments), or SE15 (for clade A/B alignments) as reference genomes. IS elements and repeat regions (>95% identity) detected in the reference genomes with ISEScan v1.7.2.3^48^ (default options) and NUCmer v3.1^49^ (maxmatch and nosimplify options), respectively, were masked in the alignment. Recombination-associated SNPs were filtered out using gubbins v2.4.1^50^ (default parameters). Maximum-likelihood phylogenetic trees were generated using IQ-TREE v2.0.3^51^ with the generalized time-reversible (GTR) model and gamma distribution with 100 bootstraps to assess confidence. The recombinant-free SNP alignment was passed to IQ-TREE together with the number of invariant sites (fconst option) of each nucleotide in the core genome alignment, as identified using snp-sites v2.5.1 (-C flag)^52^. Alignment metrics are provided in the figure captions. Phylogenetic clusters were determined by hierarchical Bayesian analysis from SNP alignments using fastbaps v1.0.5^53^ over three levels with optimised BAPS priors. For the BAPS analysis, separate recombinant-free SNP alignments generated as described above were used for clades A/B, C1, and C2. *papGII*+ sublineages were defined as BAPS clusters consisting of at least 10 isolates of which >90% harboured *papGII*.

### AMR-conferring gene content

Pointfinder v3.1.0^54^ was used to identify chromosomal mutations in QRDRs. Ciprofloxacin resistance was here predicted based on the presence of plasmid-mediated quinolone resistance genes or at least four amino acid changes associated with quinolone resistance in GyrA (S83L, S83A, D87G, D87N, D87Y), ParC (S57T, S80I, S80R, E84G, E84V, E84K), or ParE (L445H, S458A, E460D, I529L). Identified mutations are listed in Supplementary Data 4. ARGs were identified using ABRicate v0.9.3^55^ in conjunction with the resfinder database^56^ (minimum sequence coverage/identity 70%/90%). Network graphs were constructed in R 3.5.0 for ARG combinations co-occurring in at least 15 isolates with a Jaccard distance of <0.5. For each pair of ARGs (ARG_A_ and ARG_B_), Jaccard distance (1–|ARG_A_∩ARG_B_|/|ARG_A_ ∪ ARG_B_|) were calculated with the vegdist function in the R package vegan v2.5-7^57^ and networks were analysed and visualized using the R package igraph v1.2.6^58^. The genetic context of *bla*CTX-M genes was inspected manually using CLC sequence viewer 8. Disruptions of the *mppA* and *DUF4132* loci were investigated by determining their BLAST alignment coverage using ABRicate v0.9.3 with *mppA* (P423_RS08085) and the *DUF4132* region (P423_RS12390–P423_RS12392) from strain JJ1886 (GCF_000493755.1) as query. Hits with <90% (*mppA*) and <70% (*DUF4132* region) query coverage were classified as disrupted.

### Identification of virulence genes, plasmid replicons, and ST131 clade affiliation

Virulence-associated genes including *papGII* were identified using ABRicate v0.9.3 (minimum sequence coverage/identity 70/90%) in conjunction with the EcVGDB database^59^. Assemblies that contained *papGII* (>70% sequence coverage, >98% sequence identity) were defined as *papGII*+ isolates. IncF family replicon alleles were identified using the pubMLST RESTful API v1.27.0^60^ (IncF RST scheme) and replicon types of other families with ABRicate v0.9.3^55^ in conjunction with the plasmidfinder database^61^ (minimum sequence coverage/identity 70%/90%). Isolates were assigned to clades based on phylogenetic clustering. Clade assignment was supported by the presence of QRDR mutations, *fimH* types identified using FimTyper v1.1^62^, and the phylogenetic distribution of previously typed isolates. Genome assemblies were annotated using Prokka v1.13.3^63^. Comparisons of genomic regions were created using EasyFig v2.2.3^64^ and processed in Inkscape v0.92. *papGII*+ PAI types were determined by calculating mash distances to reference PAIs using mashtree v1.2.0^65^ and hierarchical clustering (UPGMA) in R v4.0.3 with a distance cut-off of 0.04, as described previously^11^.

### Statistical tests

Statistical analyses were performed using R version 3.5.3. Frequency counts were compared using a two-tailed Fisher’s exact test, while non-normally distributed continuous variables were analysed using the Mann-Whitney U test (two-sided). *P* values were adjusted for multiple testing using Bonferroni correction and adjusted *P* values of <0.05 were considered to reflect statistical significance.

### Reporting summary

Further information on research design is available in the Nature Research Reporting Summary linked to this article.

## Supplementary information

Supplementary Information

Description of Additional Supplementary Files

Supplementary Data 1

Supplementary Data 2

Supplementary Data 3

Supplementary Data 4

Reporting Summary

## Data Availability

All genome assemblies were obtained from public databases. Accession numbers are listed in Supplementary Data 1 (main dataset) and Supplementary Data 3 (validation dataset). Source data for the main figures and calculations can be found in Supplementary Data 1, 2 and 3. All other data are available from the corresponding author on reasonable request.
