# Supplementary material for: Convergence of virulence and antimicrobial resistance in increasingly prevalent Escherichia coli ST131 papGII+ sublineages
Source: Commun Biol. 2022 Jul 28;5:752. doi: 10.1038/s42003-022-03660-x (PMC9334617; doi:10.1038/s42003-022-03660-x)
Supplement: Supplementary file 1 — Supplementary Information [file 42003_2022_3660_MOESM1_ESM.pdf]

Supplementary Tables and Figures

Convergence of virulence and antimicrobial resistance in increasingly prevalent *Escherichia coli* ST131 *papGII*+ sublineages

Michael Biggel,[1,#]*, Pieter Moons[1], Minh Ngoc Nguyen[1], Herman Goossens[1], Sandra Van Puyvelde[1,2]*

[1] Laboratory of Medical Microbiology, Vaccine & Infectious Disease Institute, University of Antwerp, Antwerp, Belgium

[2] Cambridge Institute of Therapeutic Immunology & Infectious Disease (CITIID), Department of Medicine, University of Cambridge, Cambridge, United Kingdom

[#] Current address: Institute for Food Safety and Hygiene, Vetsuisse Faculty, University of Zurich, Zurich, Switzerland

* Corresponding authors

Email: michael.biggel@uzh.ch (MB), Sandra.VanPuyvelde@uantwerpen.be (SVP)

Supplementary Table 1: Prevalence of *bla*CTX-M alleles by clade affiliation.

Clade	No. isolates	Prevalence (%)				
		***bla*CTX-M-1**	***bla*CTX-M-14**	***bla*CTX-M-15**	***bla*CTX-M-27**	***bla*CTX-M-101**
Clade A	174	12 (6.9%)	23 (13.2%)	39 (22.4%)	28 (16.1%)	0 (0%)
Clade B	126	4 (3.2%)	3 (2.4%)	5 (4%)	0 (0%)	27 (21.4%)
Clade C0	19	0 (0%)	0 (0%)	5 (26.3%)	0 (0%)	0 (0%)
Clade C1	406	1 (0.2%)	65 (16%)	30 (7.4%)	188 (46.3%)	0 (0%)
Clade C2	913	0 (0%)	4 (0.4%)	811 (88.8%)	0 (0%)	0 (0%)

Supplementary Table 2: Characterization of resolved *papGII*+ PAIs identified in 29 high-quality genome assemblies.

Strain ID	ST131 clade	*papGII*+ sublineage	*papGII*+ PAI insertion site	*papGII*+ PAI type	ARGs on *papGII*+ PAI
A17EC0155	clade A	A	tRNA-*pheU*	Type III	-
AR_0089	clade B	-	tRNA-*pheV*	Not identified; *pap* disrupted	*sul2, tet(B), aph(3')-Ia, aph(6)-Id, aph(3'')-Ib*
222A118	clade C1	C1	tRNA-*pheU*	Type III	-
AR_0378	clade C2	C2 L1b	tRNA-*pheU*	Type III	-
B1017-PB	clade C2	-	tRNA-*pheU*	Type III	-
B1033-PB	clade C2	-	tRNA-*pheU*	Type III	-
B1131-PB	clade C2	C2 L1a	tRNA-*pheU*	Type III	-
B1316-PB	clade C2	-	tRNA-*pheU*	Type III	-
B1320-PB	clade C2	-	tRNA-*pheU*	Type III	-
B1323-PB	clade C2	-	tRNA-*pheU*	Type III	-
B1370-PB	clade C2	C2 L1b	tRNA-*pheU*	Type III	-
C0014-PB	clade C2	C2 L1a	tRNA-*pheU*	Type III	-
C0107-PB	clade C2	-	tRNA-*pheU*	Type III	-
C0134-PB	clade C2	C2 L1a	tRNA-*pheU*	Type III	-
EC_2_0	clade C2	C2 L2	*gln*	Type IV	-
EC_4_0	clade C2	-	tRNA-*pheU*	Type III	-
EC0_56	clade C2	C2 L1a	tRNA-*pheU*	Type III	-
EC0_76	clade C2	-	tRNA-*pheU*	Type III	-
EC1_50	clade C2	C2 L1a	tRNA-*pheU*	Type III	-
EC1_6	clade C2	-	tRNA-*pheU*	Type III	-
Ecol_656	clade C2	-	tRNA-*pheU*	Type III	-
Ecol_AZ146	clade C2	C2 L1a	tRNA-*pheU*	Type III	-
ESBL41	clade C2	C2 L3	tRNA-*pheV*	Type III	-
FDAARGOS_142	clade C2	C2 L1b	tRNA-*pheU*	Type III	-
O25b_H4	clade C2	-	tRNA-*pheU*	Type III	-
p11A	clade C2	C2 L1a	tRNA-*pheU*	Type III	-
p4A	clade C2	C2 L1a	tRNA-*pheU*	Type III	-
S65EC	clade C2	-	tRNA-*pheU*	Type III	-
US02	clade C2	C2 L1a	tRNA-*pheU*	Type III	-

PAI: pathogenicity island; ARGs: acquired antimicrobial resistance genes

Supplementary Table 3: Average number of antimicrobial resistance genes (ARGs) among 1,538 ST131 isolates from the 11 source collections by *papGII* status and isolate characteristics.

	No. *papGII*-negative isolates	No. *papGII*+ isolates	Average no. ARGs (SD; median)		P_{adj} [1]	Odds ratio [2] (95 % Confidence interval)
			papGII-negative isolates	*papGII*+ isolates		
Overall [3]	1020	518	6.2 (SD 3.7; 7)	8.7 (SD 3.6; 9)	<0.001	2.2 (1.8 - 2.7)
Clade affiliation						
Clade A	133	26	5.4 (SD 4.0; 5)	8.1 (SD 4.3; 11)	0.02	4.7 (1.9 - 11.7)
Clade B	76	38	2.9 (SD 3.3; 2)	11.4 (SD 2.9; 13)	<0.001	79.7 (21.8 - 292.1)
Clade C1	346	36	6.0 (SD 3.9; 8)	7.8 (SD 3.6; 9)	0.01	1.3 (0.6 - 2.6)
Clade C2	447	418	7.1 (SD 3.2; 8)	8.5 (SD 3.5; 9)	<0.001	1.7 (1.3 - 2.2)
Year of isolation						
2001 - 2008	127	12	6.4 (SD 3.6; 7)	11.3 (SD 4.4; 12)	0.002	9.1 (1.9 - 43.4)
2009 - 2011	243	88	6.2 (SD 3.8; 7)	9.3 (SD 4.2; 10)	<0.001	2.9 (1.7 - 4.8)
2012 - 2014	419	208	6.2 (SD 3.6; 7)	8.3 (SD 3.4; 9)	<0.001	1.6 (1.1 - 2.2)
2015 - 2017	231	210	5.9 (SD 3.9; 7)	8.6 (SD 3.4; 9)	<0.001	2.5 (1.7 - 3.7)
Collection pre-selected for ESBL-producers						
no	314	76	5.1 (SD 3.7; 5)	7.7 (SD 4.0; 9)	<0.001	2.9 (1.7 - 4.8)
yes	706	442	6.6 (SD 3.6; 8)	8.9 (SD 3.5; 9)	<0.001	1.8 (1.4 - 2.3)
Clinical source						
feces	52	21	6.9 (SD 3.1; 7.5)	8.4 (SD 3.8; 9)	0.7	2.0 (0.7 - 5.8)
urine	161	136	7.3 (SD 3.8; 9)	9.3 (SD 3.8; 10)	<0.001	1.7 (1.1 - 2.8)
blood	530	292	5.2 (SD 3.7; 5)	8.6 (SD 3.6; 9)	<0.001	3.5 (2.6 - 4.7)

[1] Mann-Whitney U test, Bonferroni adjusted; [2] odds ratio of harbouring>8 ARGs; [3] including assemblies of clade C0, unknown year of isolation, and unknown/other clinical sources

SD: standard deviation; ESBL: extended-spectrum beta-lactamase

Supplementary Table 4: Average number of antimicrobial resistance genes (ARGs) by clinical source and presence of *papGII* among 1,638 ST131 isolates from the main dataset combined with 3,608 assemblies of human clinical isolates from EnteroBase (validation dataset, Supplementary Data 3).

Clinical source	No. *papGII*-negative isolates	No. *papGII*+ isolates	Average no. ARGs (SD; median)		P_{adj} [1]	Odds ratio [2] (95 % Confidence interval)
			papGII-negative isolates	*papGII*+ isolates		
Faeces	507	89	5.9 (SD 3.9; 6)	8.1 (SD 3.1; 9)	<0.001	1.8 (1.2 - 2.9)
Urine	855	397	6.5 (SD 3.9; 7)	8.4 (SD 3.5; 9)	<0.001	1.9 (1.5 - 2.4)
Blood	1,023	615	5.9 (SD 3.8; 6)	8.3 (SD 3.4; 9)	<0.001	2.1 (1.7 - 2.6)
Overall [3]	3,752	1,494	6.1 (SD 3.8; 7)	8.3 (SD 3.5; 9)	<0.001	1.8 (1.6 - 2)

[1] Mann-Whitney U test, Bonferroni adjusted; [2] odds ratio of harbouring >8 ARGs; [3] including assemblies of 1,760 isolates from other/unknown clinical sources

SD: standard deviation

Supplementary Table 5: Antimicrobial resistance genes (ARGs) significantly (P_{adj} < 0.05; Fisher's exact test, Bonferroni corrected for the overall number of identified ARGs [n = 102]) associated with *papGII*-positive (*papGII*+) versus *papGII*-negative isolates by clade affiliation.

(a) Clade A

ARG	Resistance class	Prevalence *papGII*+ isolates (n = 27)	Prevalence *papGII*-negative isolates (n = 147)	Odds ratio (95 % CI)
*bla*CTX-M-27	Beta-lactam (ESBL)	22 (81.5%)	6 (4.1%)	103.4 (29.1 – 367.8)

(b) Clade B

ARG	Resistance class	Prevalence *papGII*+ isolates (n = 39)	Prevalence *papGII*-negative isolates (n = 87)	Odds ratio (95 % CI)
aac(3)-IId	Aminoglycosides	36 (92.3%)	13 (14.9%)	68.3 (18.3 – 254.9)
aadA2	Aminoglycosides	35 (89.7%)	11 (12.6%)	60.5 (18.0 – 203.2)
aph(3'')-Ib	Aminoglycosides	30 (76.9%)	12 (13.8%)	20.8 (8.0 – 54.5)
aph(6)-Id	Aminoglycosides	30 (76.9%)	14 (16.1%)	17.4 (6.8 – 44.5)
*bla*CTX-M-101	Beta-lactam (ESBL)	26 (66.7%)	1 (1.1%)	172 (21.5 – 1377.8)
*bla*TEM-1B	Beta-lactam	33 (84.6%)	24 (27.6%)	14.4 (5.4 – 38.8)
catA1	Chloramphenicols	33 (84.6%)	2 (2.3%)	233.4 (44.9 – 1217.2)
dfrA12	Trimethoprim	35 (89.7%)	9 (10.3%)	75.9 (21.9 – 263.0)
floR	Chloramphenicols	25 (64.1%)	3 (3.4%)	50.0 (13.3 – 188.0)
mph(A)	Macrolides	36 (92.3%)	13 (14.9%)	68.3 (18.3 – 254.9)
sul1	Sulphonamides	35 (89.7%)	19 (21.8%)	31.3 (9.9 – 99.2)
sul2	Sulphonamides	30 (76.9%)	16 (18.4%)	14.8 (5.9 – 37.2)
tet(A)	Tetracyclines	25 (64.1%)	20 (23.0%)	6.0 (2.6 – 13.6)

(c) Clade C1

ARG	Resistance class	Prevalence *papGII*+ isolates (n = 37)	Prevalence *papGII*-negative isolates (n = 369)	Odds ratio (95 % CI)
aac(3)-IId	Aminoglycosides	23 (62.2%)	69 (18.7%)	7.1 (3.5 – 14.6)
*bla*CTX-M-14	Beta-lactam (ESBL)	27 (73.0%)	38 (10.3%)	23.5 (10.6 – 52.3)
*bla*CTX-M-27	Beta-lactam (ESBL)	6 (16.2%)	182 (49.3%)	0.20 (0.08 – 0.48)
*bla*TEM-1B	Beta-lactam	29 (78.4%)	150 (40.7%)	5.3 (2.4 – 11.9)

(d) Clade C2

ARG	Resistance class	Prevalence *papGII*+ isolates (n = 444)	Prevalence *papGII*-negative isolates (n = 469)	Odds ratio (95 % CI)
aac(3)-IIa	Aminoglycosides	235 (52.9%)	79 (16.8%)	5.6 (4.1 – 7.5)
aac(3)-IId	Aminoglycosides	59 (13.3%)	18 (3.8%)	3.8 (2.2 – 6.6)
aac(6')-Ib-cr	Aminoglycosides, Fluoroquinolones	371 (83.6%)	307 (64.5%)	2.7 (2.0 – 3.7)
aph(3'')-Ib	Aminoglycosides	109 (24.5%)	45 (9.6%)	3.1 (2.1 – 4.5)
aph(6)-Id	Aminoglycosides	107 (24.1%)	43 (9.2%)	3.1 (2.1 – 4.6)
*bla*CTX-M-15	Beta-lactam (ESBL)	427 (96.2%)	384 (81.9%)	5.6 (3.2 – 9.5)
*bla*OXA-1	Beta-lactam	370 (83.3%)	312 (65.5%)	2.5 (1.8 – 3.4)
catA1	Chloramphenicols	45 (10.1%)	9 (1.9%)	5.8 (2.8 – 11.9)
(Δ)*catB3*	Chloramphenicols	367 (82.7%)	306 (65.2%)	2.5 (1.9 – 3.5)
dfrA14	Trimethoprim	34 (7.7%)	6 (1.3%)	6.4 (2.7 – 15.4)
sul2	Sulphonamides	109 (24.5%)	44 (9.4%)	3.1 (2.2 – 4.6)
tet(B)	Tetracyclines	51 (11.5%)	18 (3.8%)	3.3 (1.9 – 5.7)

Supplementary Figures

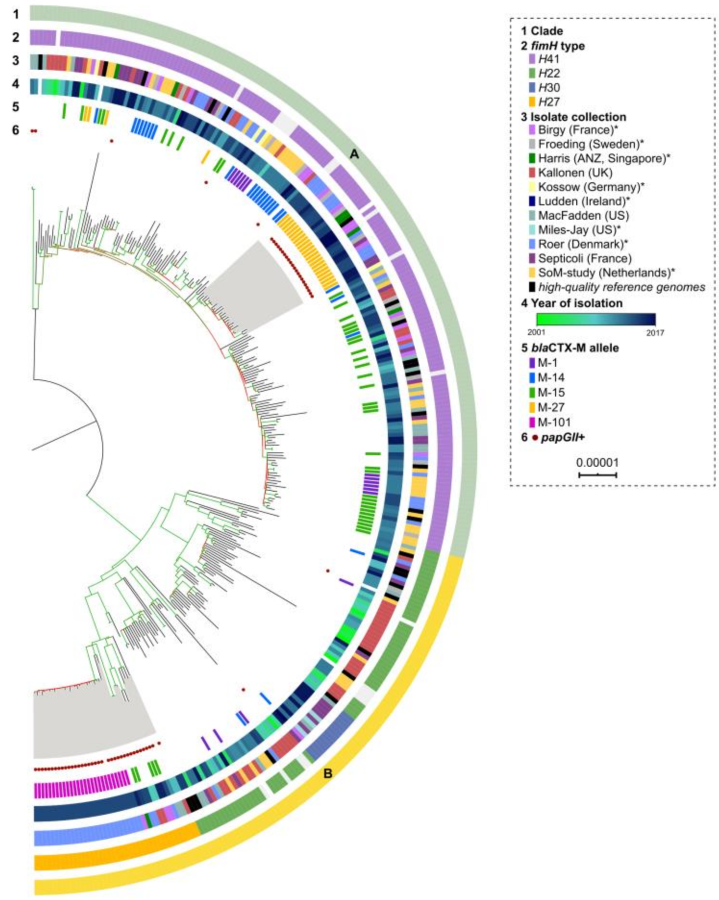

Supplementary Fig. 1. Phylogenetic tree of ST131 clades A and B. Maximum-likelihood phylogenetic tree of 300 isolates of clade A and B. The tree is based on 5,206 variable sites in a 3.3 Mb core genome alignment. Each isolate is annotated with ST131 clade affiliation (ring 1), *fimH* type (ring 2), source collection (ring 3), year of isolation (ring 4), *bla*CTX-M allele (ring 5), and presence of *papGII* (ring 6). Collections that consisted only of ESBL isolates are labelled with an asterisk in the legend. For isolates that were annotated with the isolation time period instead of the exact isolation year, the midrange value is shown. *papGII+* sublineages discussed in the text are shaded in grey. Colours of branches indicate confidence intervals (gradient from green [bootstrap 100] to red [bootstrap 0]). The scale bar indicates the number of substitutions per core genome alignment site. The tree was visualized using iTOL [1]. ANZ: Australia and New Zealand

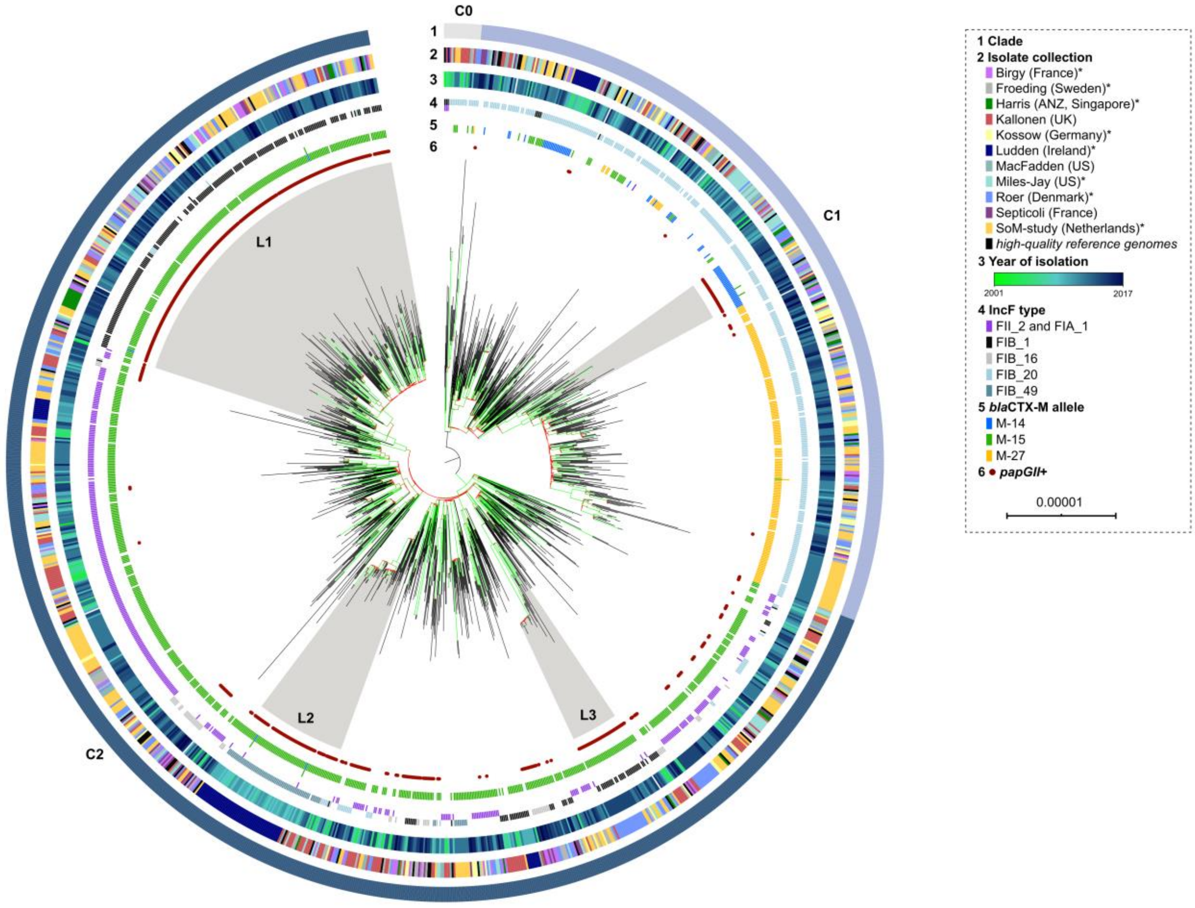

Supplementary Fig. 2. Phylogenetic tree of ST131 clades C0, C1, or C2. Maximum-likelihood phylogenetic tree of 1,338 clade C isolates based on 10,904 variable sites in a 2.5 MB core genome alignment. Each isolate is annotated with ST131 subclade affiliation (ring 1), source collection (ring 2), year of isolation (ring 3), plasmid replicon type (only selected IncF pMLST types shown; ring 4), *bla*CTX-M allele (ring 5), and presence of *papGII* (ring 6). Collections that consisted only of ESBL isolates are labelled with an asterisk in the legend. For isolates that were annotated with the isolation time period instead of the exact isolation year, the midrange value is shown. *papGII*+ sublineages discussed in the text are shaded in grey. Those belonging to clade C2 are annotated with L1, L2, and L3. The scale bar indicates the number of substitutions per core genome alignment site. Colours of branches indicate confidence intervals (gradient from green [bootstrap 100] to red [bootstrap 0]). The tree was visualized using iTOL [1]. ANZ: Australia and New Zealand

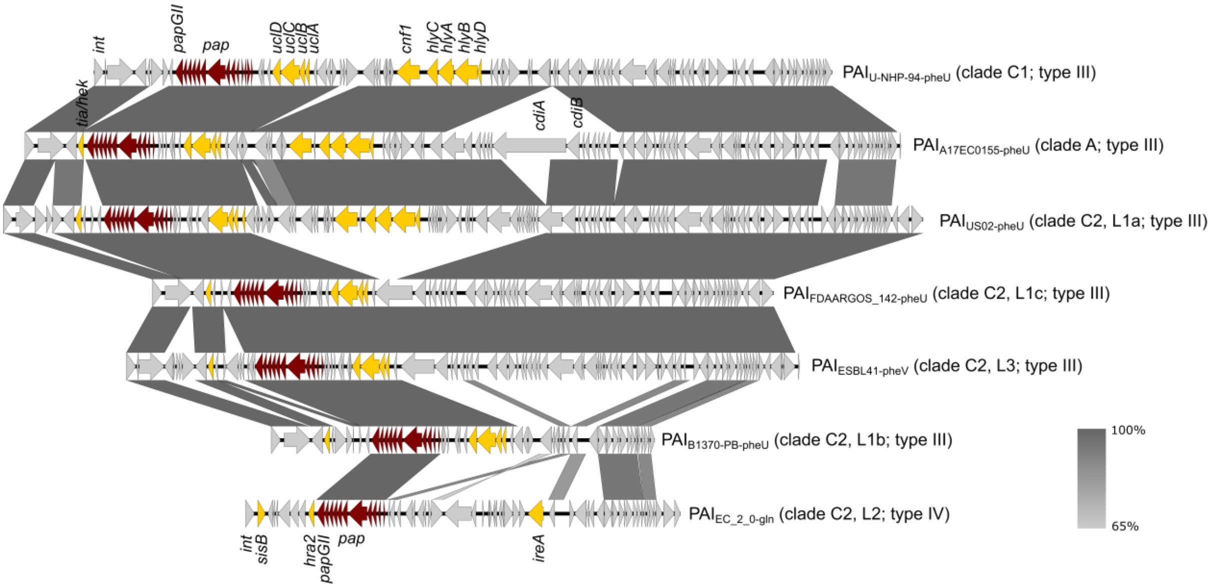

Supplementary Fig. 3: *papGII*-containing pathogenicity islands (PAIs) in high-quality assemblies of isolates from *papGII*+ sublineages of clades A, C1, and C2. $\text{PAI}_{\text{EC\_2\_0-gln}}$ belongs to the type IV PAI, whereas all other islands were identified as type III PAIs. Grey shaded boxes between sequences indicate homologous regions. The level of nucleotide identity is given in the scale bar. The *papGII* operon and other virulence genes are coloured. The figure was created in Easyfig v2.1 [2].

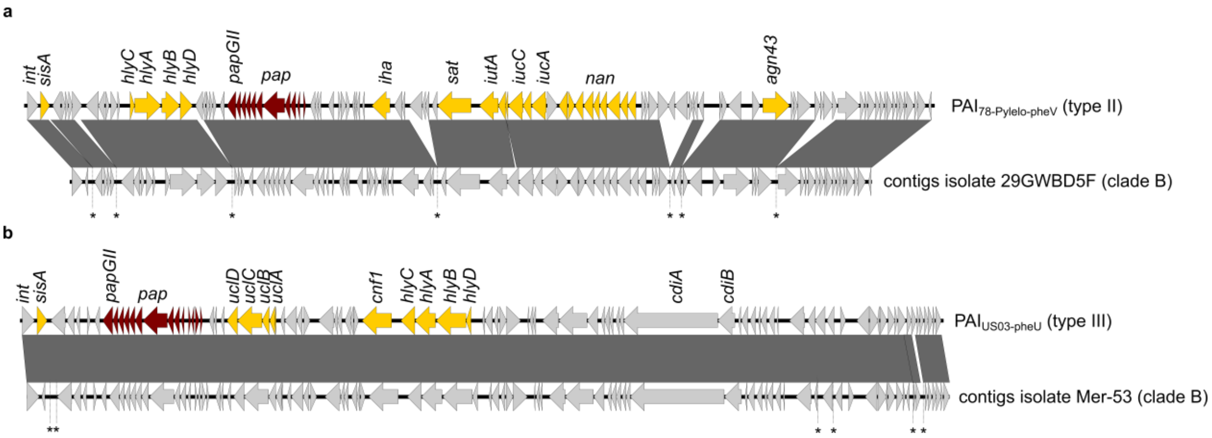

Supplementary Fig. 4: Putative *papGII*-containing pathogenicity islands (PAIs) in two ST131 isolates belonging to the *papGII*+ sublineage in clade B. (a) Assembly contigs of isolate 29GWBD5F (ESC_GA4904AA) compared to a type II *papGII*+ PAI (isolate 78-Pyelo, GCA_014131615.1) and (b) contigs of MER-53 (ESC_GA9528AA) compared to a type III *papGII*+ PAI (isolate US03, GCA_014131595.1). Contig breaks are indicated with asterisks. Grey shaded boxes between sequences indicate homologous regions with >95 % nucleotide identity. The *pap* operon and other virulence genes are coloured. The figure was created in EasyFig v2.1 [2].

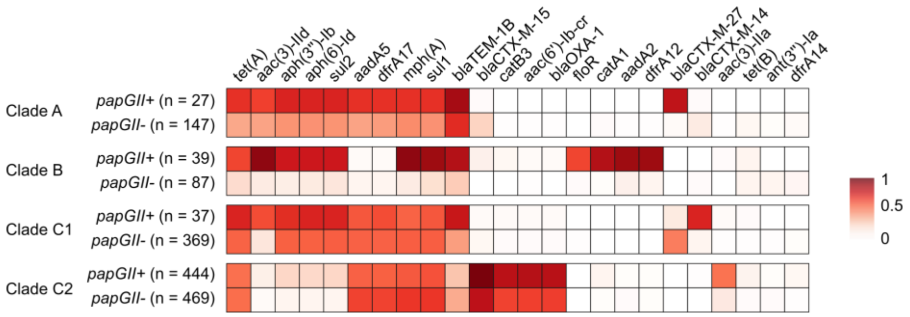

Supplementary Fig. 5: Prevalence of acquired antimicrobial resistance genes (ARGs) by clade affiliation and *papGII* presence. ARGs occurring in >30 isolates are shown. The prevalence of ARGs is given according to the scale bar.

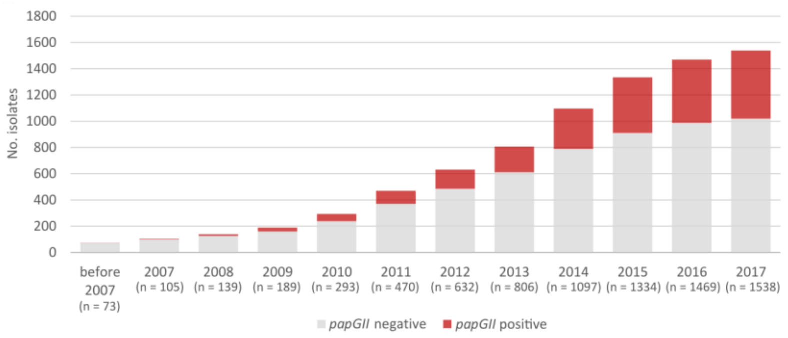

Supplementary Fig. 6: Cumulative number of *papGII*-containing isolates in the ST131 population over time. 1,538 isolates from the 11 investigated collections were analysed. The cumulative proportion of *papGII*-containing isolates is coloured in red. The cumulative total number of isolates is given in brackets.

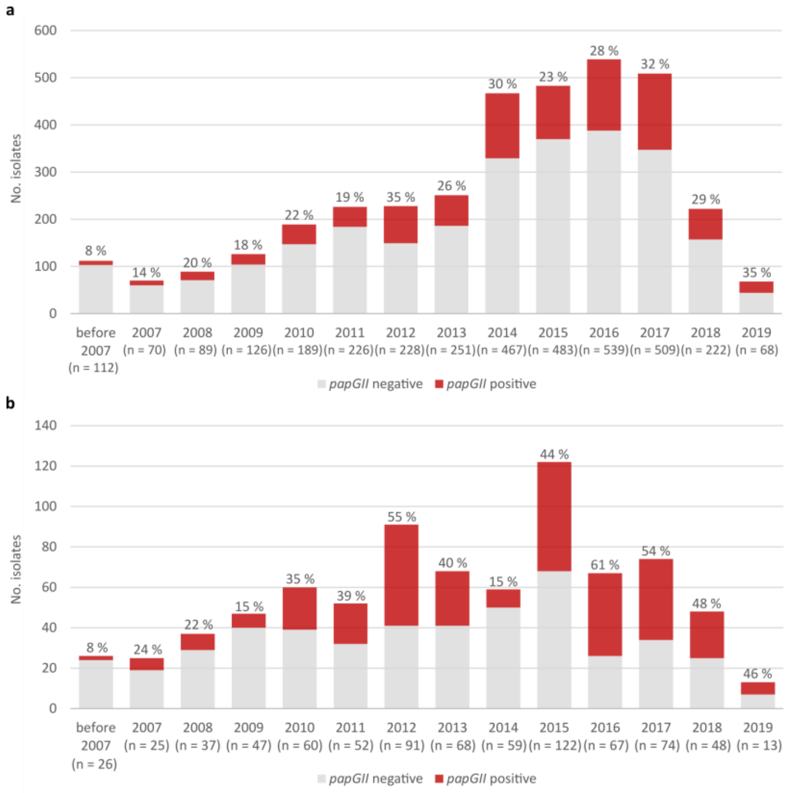

Supplementary Fig. 7: Proportion of *papGII*-containing isolates over time among assemblies of human ST131 isolates available on EnteroBase. (a) Prevalence of *papGII* among 3,608 assemblies of human ST131 isolates. (b) Prevalence of *papGII* among 795 assemblies of ST131 isolates labelled as human blood or bacteremia isolates. The percentage of *papGII*-containing isolates per year is indicated. Assemblies included in the main dataset were excluded. The total number of isolates per year is shown in brackets (x-axis).

Supplementary References

1. Letunic, I. & Bork, P. Interactive Tree Of Life (iTOL) v4: recent updates and new developments. *Nucleic Acids Res.* **47**, W256–W259 (2019).

2. Sullivan, M. J., Petty, N. K. & Beatson, S. A. Easyfig: A genome comparison visualizer. *Bioinformatics* **27**, 1009–1010 (2011).
