# Supplementary material for: Convergence of virulence and antimicrobial resistance in increasingly prevalent Escherichia coli ST131 papGII+ sublineages
Source: Commun Biol. 2022 Jul 28;5:752. doi: 10.1038/s42003-022-03660-x (PMC9334617; doi:10.1038/s42003-022-03660-x)
Supplement: Supplementary file 2 — Description of Additional Supplementary Files [file 42003_2022_3660_MOESM2_ESM.pdf]

Description of Additional Supplementary Files

File name: Supplementary Data 1
Description: Characteristics, metadata, and assembly metrics of bacterial isolates included in the main dataset.

File name: Supplementary Data 2
Description: Presence and absence of antimicrobial resistance genes identified in the main dataset isolates.

File name: Supplementary Data 3
Description: Characteristics, metadata, and assembly metrics of bacterial isolates included in the validation dataset.

File name: Supplementary Data 4
Description: Mutations in quinolone-resistance determining regions (QRDRs) in the main dataset isolates.
