# Supplementary material for: Convergence of virulence and antimicrobial resistance in increasingly prevalent Escherichia coli ST131 papGII+ sublineages
Source: Commun Biol. 2022 Jul 28;5:752. doi: 10.1038/s42003-022-03660-x (PMC9334617; doi:10.1038/s42003-022-03660-x)
Supplement: Supplementary file 7 — Reporting Summary [file 42003_2022_3660_MOESM7_ESM.pdf]

nature portfolio

Corresponding author(s): Michael Biggel
Sandra Van Puyvelde

Last updated by author(s): 16/6/22

Reporting Summary

Nature Portfolio wishes to improve the reproducibility of the work that we publish. This form provides structure for consistency and transparency in reporting. For further information on Nature Portfolio policies, see our Editorial Policies and the Editorial Policy Checklist.

Statistics

For all statistical analyses, confirm that the following items are present in the figure legend, table legend, main text, or Methods section.

n/a	Confirmed	
☐	☒	The exact sample size (*n*) for each experimental group/condition, given as a discrete number and unit of measurement
☒	☐	A statement on whether measurements were taken from distinct samples or whether the same sample was measured repeatedly
☐	☒	The statistical test(s) used AND whether they are one- or two-sided<br>*Only common tests should be described solely by name; describe more complex techniques in the Methods section.*
☒	☐	A description of all covariates tested
☐	☒	A description of any assumptions or corrections, such as tests of normality and adjustment for multiple comparisons
☐	☒	A full description of the statistical parameters including central tendency (e.g. means) or other basic estimates (e.g. regression coefficient) AND variation (e.g. standard deviation) or associated estimates of uncertainty (e.g. confidence intervals)
☐	☒	For null hypothesis testing, the test statistic (e.g. *F*, *t*, *r*) with confidence intervals, effect sizes, degrees of freedom and *P* value noted<br>*Give P values as exact values whenever suitable.*
☒	☐	For Bayesian analysis, information on the choice of priors and Markov chain Monte Carlo settings
☒	☐	For hierarchical and complex designs, identification of the appropriate level for tests and full reporting of outcomes
☒	☐	Estimates of effect sizes (e.g. Cohen's *d*, Pearson's *r*), indicating how they were calculated

Our web collection on statistics for biologists contains articles on many of the points above.

Software and code

Policy information about availability of computer code

Data collection	Not applicable
Data analysis	mlst v2.16.1<br>Quast v5.0.2<br>Parsnp v1.2<br>Gubbins v2.4.1<br>IQ-TREE v2.0.3<br>Pointfinder releaes 3.1.0<br>ABRicate v0.9.3<br>R 3.5.0<br>CLC Sequence Viewer 8<br>pubMLST RESTful API v1.27.0<br>FimTyper v1.1<br>Prokka v1.13.3<br>EasyFig 2.2.3<br>Inkscape 0.92<br>Mashtree v1.2.0<br>itol 5

For manuscripts utilizing custom algorithms or software that are central to the research but not yet described in published literature, software must be made available to editors and reviewers. We strongly encourage code deposition in a community repository (e.g. GitHub). See the Nature Portfolio guidelines for submitting code & software for further information.

Data

Policy information about availability of data

All manuscripts must include a data availability statement. This statement should provide the following information, where applicable:

- Accession codes, unique identifiers, or web links for publicly available datasets
- A description of any restrictions on data availability
- For clinical datasets or third party data, please ensure that the statement adheres to our policy

All genome assemblies were obtained from public databases. Accession numbers are listed in Supplementary Data 1 (main dataset) and Supplementary Data 4 (validation dataset).

Field-specific reporting

Please select the one below that is the best fit for your research. If you are not sure, read the appropriate sections before making your selection.

☐ Life sciences ☐ Behavioural & social sciences ☒ Ecological, evolutionary & environmental sciences

For a reference copy of the document with all sections, see nature.com/documents/nr-reporting-summary-flat.pdf

Ecological, evolutionary & environmental sciences study design

All studies must disclose on these points even when the disclosure is negative.

Study description	We used publicly genomes from recent global ST131 isolates to analyze evolutionary trends
Research sample	Main dataset: comprises 1,638 ST131 genomes. These include 1,538 whole-genome draft assemblies from 11 collections and 100 high-quality reference assemblies. The isolates originated from human feces, bloodstream infections, urinary tract infections, or other clinical sources, and were isolated between 2001 and 2017 in Europe, North America, Asia, and Oceania.<br><br>Validation dataset: comprises 3,608 ST131 genomes from EnteroBase and was used to confirm the increasing proportion of papGII-carrying ST131 isolates over time and the association of papGII with distinct clinical sources
Sampling strategy	Main dataset: Literature was searched for published datasets of ST131 genomes. Large (>40) single-study genome collections of well defined isolates from human sources were considered for inclusion. To investigate the genetic context of virulence and resistance genes, 100 high-quality reference assemblies were also included.<br>Validation dataset: all ST131 assemblies available on EnteroBase (accessed on 07/01/2021) with known year of isolation and recovered from human samples (according BioSample metadata) were included for analysis.
Data collection	Assembled genomes were downloaded from EnteroBase or NCBI Genbank. ST131 affiliation was confirmed by in-silico mlst.
Timing and spatial scale	Main dataset: analyzed isolates were collected between 2001 and 2017 (Table 1, Suppl Data 1)<br>Validation dataset: analyzed isolates were collected between 1990 and 2020 (Suppl Data 3)
Data exclusions	Low-quality assemblies (N50 < 40 kb) were excluded.
Reproducibility	Multiple global collections were included to ensure reproducibility across a broad spatiotemporal origin.<br><br>Results on the proportion of papGII-carrying ST131 isolates over time were confirmed using a large validation dataset from EnteroBase.<br><br>To control for confounding, data were stratified by clade affiliation, source collection, and year of isolation.
Randomization	Randomization is not applicable to this study. Isolates from various observational studies were included.
Blinding	Blinding is not applicable to this study. The population cohorts were defined by previous studies.

Did the study involve field work? ☐ Yes ☒ No

Reporting for specific materials, systems and methods

We require information from authors about some types of materials, experimental systems and methods used in many studies. Here, indicate whether each material, system or method listed is relevant to your study. If you are not sure if a list item applies to your research, read the appropriate section before selecting a response.

Materials & experimental systems

n/a	Involved in the study
☒	☐ Antibodies
☒	☐ Eukaryotic cell lines
☒	☐ Palaeontology and archaeology
☒	☐ Animals and other organisms
☒	☐ Human research participants
☒	☐ Clinical data
☒	☐ Dual use research of concern

Methods

n/a	Involved in the study
☒	☐ ChIP-seq
☒	☐ Flow cytometry
☒	☐ MRI-based neuroimaging
